# Hospital Progress and Finance

**Published:** 1923-05

**Authors:** 


### HOSPITAL PROGRESS AND FINANCE.

### THE SUSSEX COUNTY HOSPITAL CONTROVERSY.

THERE are still controversy and divided counsels in regard to the Sussex County Hospital—the report of the committee of inquiry into its affairs was summarised in our April number. Discontent is centred upon the non-publication of Sir Napier Burnett's report to the investigation committee. The Board of Management appears to believe that Sir Napier's report contains a reference favourable to itself, the disclosure of which would allay criticism. The committee of inquiry, in the person of its chairman, the Rev. E. D. L. Harvey, asserts that the report was not intended for publication, and Mr. Harvey has since declared that any want of confidence there may be arises because the public is uncertain whether the Board of Management intends really to carry out the committee of inquiry's recommendations. The controversy is doing the hospital much harm, and as Sir Napier Burnett has no objection to publication, and has even offered to summarise his very long statement, a convenient solution is at hand. In our view only publication can now end the controversy, and as its ending is of vital importance to the public, all other considerations should give way. By the time these lines appear in print, we hope that a decision in this sense will have been made.

### LORD KNUTSFORD AND CATHOLIC NURSES.

THE *Catholic Herald* having published an article stating that " at a well-known London hospital no Catholic is ever engaged for the nursing staff under the same conditions as others, and no Catholic nurse can ever rise to the position of sister," Lord Kuntsford promptly wrote for the name of the hospital referred to. The paper has replied that it is Guy's, and states that a certain Rev. A. P. Flanagan is responsible for the assertion. Denominational papers are notoriously militant, but since the paper was previously assured at Guy's that nurses are engaged simply on their merits, we trust that it will ask the priest for evidence of his assertion, or else for an apology. It is he rather than the paper that is primarily responsible, though the paper gave currency to his allegation. Lord Kuntsford appears to be familiar with the charge, and can perhaps throw light on the motive that prompted it.

### A SUCCESSFUL HOSPITAL GARDEN.

HAVING noticed in the past year that several hospitals, isolation or other, have abandoned gardening because the produce was found to be less than the cost, it is pleasant to record a successful experiment. In the spring of 1922 a hundred fruit trees were planted on ground attached to the Acton (Middlesex) Isolation Hospital. Existing trees were carefully pruned, and a scheme to crop all the land was adopted. The result has been to supply the hospital with abundant vegetables, and to prove that it can depend on home-grown produce throughout the season. Sixteen varieties of cabbage are being tried, and demonstrations in pruning have been given to discharged officers on their way to the colonies to become farmers. The actual figures are not given, which is a pity, since experience shows that the success of hospital horticulture and farming depends almost entirely on the ability of the men responsible. To engage a gardener and leave all to him usually ends in disaster. We note, therefore, that it was Dr. Thomas, the Acton Medical Officer of Health, who started the schme. He applied for expert advice to Mr. G. W. Pyman, lecturer and demonstrator to the Middlesex Education Committee, who has reported the experiment to the Health Committee. We observe also that a local councillor has taken an enthusiastic interest in the garden. The gardener, Mr. Hollaway, has had valuable help in many directions. Without the accounts, the result must still be regarded with caution, but the moral is plain that a hospital garden, like any other hospital department, cannot run itself, and needs regular and expert supervision.

### SIR NAPIER BURNETT ON ANNUAL REPORTS.

WHY, asked Sir Napier Burnett at the annual court of the Royal Free Hospital, are annual reports so dead and uninteresting ? He read the majority of hospital reports and found them dull, but he believed the time would come when hospitals would have to issue quarterly or half-yearly reports of some of the interesting phases of their work. All discoveries, like that of insulin, were interesting, and each hospital where one occurred should tell the public of what had happened in " our hospital." His medical colleagues and himself appealed to them occasionally to write a special report on matters of interest to the public. It will probably be replied that annual reports are necessarily full of routine matters, and that interesting points quickly find their way into the papers. But this does not imply that more might not be heard of them, whether or not hospital reports are the best means of making them known. For those hospitals which issue magazines for their supporters, the solution should be simple. But such magazines are not yet the rule.

### THE TAXATION OF BETTING.

THIS question, which has come to the front again, less in the hope of thwarting a human instinct than as a means of raising revenue, has now reached the stage in which the practical means of taxing bets is the principal discussion. It is of interest to institutional workers because of the supplementary suggestion that the proceeds, or part of them, should be used for the benefit of hospitals. Sir Walter Gilbey, who has been racing for many years, is in favour of the plan and believes it possible. In a recent interview with the *Daily Telegraph*, he said that New South Wales raised £600,000 by means of taxes on betting in 1920-21, and estimated that at least £5,000,000 would be available for the London hospitals alone were it adopted in the infinitely larger population of this country. He was strongly opposed to the use of the revenue from this tax by the National Exchequer. While it is probably true that the devotion to charity of the proceeds of any contentious tax makes the tax less unpopular, regular State-aid is a doubtful means of supporting the voluntary system; and the employment of such a tax as that proposed might even alienate such hospital supporters as the Sunday fund, many of whom take a stern view of all betting. Meanwhile a Select Committee of the House of Commons has been appointed to consider the proposal.

### THE POST-WAR PROBATIONER.

MR. GEORGE PRIESTMAN, chairman of the Board of the Bradford Royal Infirmary, speaks of the difficulty of finding suitable candidates for the nursing profession, a difficulty which he says is growing greater. The applicants, he records, are not of as high a standard as they used to be, nor are they as well educated. The young gentlewomen who did fine work before and during the war are now to seek. Mr. Priestman thinks that nursing is becoming less fashionable. He is at a loss to explain falling off, which, if true, deserves study, for until the reason is known it is next to impossible to remedy it. Can our readers throw any light on a matter that affects all hospitals in their degrees?

### A WESTERN GROUPING SYSTEM.

ON the suggestion of Mr. H. N. Crouch, Hon. Secretary of the Somerset Voluntary Hospitals Committee, a conference of hospital representatives of Bristol, Somerset, Gloucestershire and Wilts. is being held to discuss the regrouping of the three counties into appropriate hospital units, with all the arrangements, financial and administrative, that would be needed for an efficient and well-distributed hospital service.

### THE ABOLITION OF RECOMMENDS.

THE ticket system has been abolished at the Royal Portsmouth Hospital, though the privileges of existing life subscribers who do not wish to waive their rights remain unprejudiced. The Beckett Hospital at Barnsley is considering a similar change, and one hopes that "recommends" will soon be a thing of the past. They cause so much trouble and suffering to patients in the often vain search for them, they are so often wasted by those to whom they belong, that they are more characteristic of a State-run institution than of a voluntary hospital. So long as they endure opponents of the voluntary system will always be able to say that a voluntary hospital is not free from red-tape and inhuman regulations.

### REPAIRS BY UNEMPLOYED.

THE offices of the Winter Distress League have been moved to Devonshire House, Piccadilly. The League has found employment during the past three months for seventy men on repairs and cleaning in institutions where no funds are available to pay for them under a scheme formed by the Finance and General Purposes Committee, the chairman of which is Mrs. H. F. Wood. The materials are paid for or supplied by the officials of each institution, and the men, who receive trades union wages, are supplied by the League. A guarantee is always given that the work would not be undertaken except by this means. Thus hospitals and unemployed ex-Service men are both helped. The London, Great Northern, Middlesex and St. Thomas's Hospitals have all made use of the plan. The League also sends women helps into the homes of the poor where the mother is ill. The six women thus employed do not do any work or nursing that would otherwise be paid for. Under-nourished children whose fathers are unemployed, are also boarded out, and clothing is supplied to unemployed whose shabby appearance would prevent their engagement. We shall watch with interest to see how far the scheme can grow. It is an interesting experiment.

